# Psychiatric symptoms in adult patients with cerebral palsy: A cohort study

**DOI:** 10.3389/fneur.2022.998922

**Published:** 2022-09-27

**Authors:** Silvia Pizzighello, Marianna Uliana, Martina Michielotto, Alda Pellegri, Matteo G. F. Vascello, Sara Piccoli, Michela Martinuzzi, Andrea Martinuzzi

**Affiliations:** ^1^Department of Conegliano, Scientific Institute IRCCS E. Medea, Treviso, Italy; ^2^Department of Bosisio Parini, Scientific Institute, IRCCS E. Medea, Lecco, Italy; ^3^Clinical Psychology Unit, ASST Papa Giovanni XXIII Hospital, Bergamo, Italy; ^4^Department of Mental Health, AULSS 6 Euganea, Padua, Italy; ^5^King's College London GKT School of Medical Education, London, United Kingdom

**Keywords:** cerebral palsy, adult patients, psychiatric symptoms, mental health, cohort study

## Abstract

**Background:**

Patients with cerebral palsy (CP) have an increased risk of developing mental health disorders.

**Aims:**

This paper is aimed to investigate the occurrence of psychiatric symptoms in adults with CP and to explore the relation between clinical and psychosocial variables.

**Methods and procedures:**

We included 199 adults with a diagnosis of CP. The chi-square and the Mann-Whitney U tests were used to compare clinical and psychosocial variables, the level of perceived disability, and the type of observed parental style in patients with and without psychiatric symptoms. Logistic regression analysis was used to identify variables that could predict the occurrence of mental health disorders.

**Outcome and results:**

Anxiety and psychosis were the most represented disorders. Age, living status, assumption of drugs, motor, manual, and global impairment were significantly different between patients with and without psychiatric symptoms. Similarly, a different parental style was observed between the two groups. Logistic regression indicated that living status, prescribed drugs, parental style, and the perceived disability in getting along with others predicted the occurrence of psychiatric symptoms.

**Conclusions and implications:**

Results suggest that patients with and without psychiatric symptoms have different clinical and psychosocial characteristics. Some variables should be considered as potentially affecting the mental health of patients with CP.

## Introduction

Cerebral palsy (CP) is an umbrella term that describes a group of permanent disorders of the development of movement and posture, which are attributed to non-progressive disturbances that occur in the developing fetal or infant brain (1). Children and adolescents with chronic conditions, such as individuals with CP, have an increased risk of developing mental health disorders due to physical and social characteristics (2, 3). Co-occurring factors, such as sensory impairment, epilepsy, sleep disturbances, poor physical activity, and pain, represent risk factors for mental health that are often observed in individuals with CP (3, 4).

On the basis of the extension of the impairment, there are different types of CP: diplegia when both legs are affected but arms could be less impaired, hemiplegia when one side of the body is affected, and quadriplegia when both legs and arms are affected.

Several studies examined the prevalence of mental health disorders in children with CP (2–8). Results showed an increased occurrence of depression, anxiety, behavior/conduct problems, and multimorbidity (two or more disorders). The role of gender, age, type and severity of CP, intellectual disability (ID), and living status on the occurrence of mental health problems remains unclear (2, 5–7). However, physical activity, sleep duration, and pain have been observed to account for some association between CP and mental health disorders, in particular, for depression and multimorbidity (3).

Furthermore, in the adult CP population, studies showed a higher prevalence of all mental disorders (9–11); in particular, these patients have an increased risk for anxiety and depression (10, 12–17).

The role of several factors on the mental health of patients with CP has been studied. For example, Van der Sloot (12) found an association of the level of motor impairment measured with the Gross Motor Function Classification System—Expanded & Revised (GMFCS-E&R) (18) but no difference based on sex. Other studies found that the occurrence of other impairments, such as epilepsy, gastrointestinal, or respiratory disorders, seemed to be unrelated (13, 16) while the influence of a concurrent ID on mental health remained controversial (11, 13). Whitney et al. (17, 19) found that several factors, as sleep disorders, pain and fatigue, were associated with different prevalence of mental health disorders.

McMorris et al. (11) suggested different reasons for the observed worse mental health in patients with CP as the occurrence of physiological and physical difficulties and the subsequent limitation on day-to-day functioning.

Contrary to these research studies, Schmidt et al. (20) found general equal levels of mental health in young and adult patients with CP when compared to healthy controls. They suggested the occurrence of a “disability paradox” that showed the higher capacity of patients to tolerate and adapt to adversity better than healthy people.

In the light of this debate, this cohort study was designed to gain more insight into the psychiatric symptoms in adult patients affected by CP. The collected data included functional scales, clinical interviews, and self-report questionnaires.

This paper is aimed:

- to explore the occurrence of psychiatric symptoms in adult patients with CP;
- to compare the clinical and the psychosocial characteristics of patients with psychiatric disorders (*Psych group*) with patients without psychiatric disorders (*No psych group*); and
- to explore the predictors of the occurrence of psychiatric disorders in adults with CP.

## Materials and methods

### Patients

Patients were enrolled in the study between 2018 and 2019 at one of the Italian Children Rehabilitation Networks in the Northeastern Italian Region of Veneto. This network provides pediatric care, and patients are discharged once they reach 18 years of age; all the patients included in the study were over 18 and had been discharged from our rehabilitation centers due to the age limit. Inclusion criteria were as follows: (1) diagnosis of CP; (2) first contact date of the patients with the healthcare service between 1985 and 2015; (3) duration of in-charge ≥3 years; and (4) age at the time of interview between 25 and 50 (i.e., year of birth between June 1967 and June 1997). The sample included 199 participants (120 men, 79 women), and the median age at the time of the interview was 32.0 [confidence interval (CI) 24.5–42, range min–max 20–50]. The study has been reviewed and approved by the Ethics Committee (Prot. N. 61/17-CE). It is adherent to the committee's recommendations and was conducted in accordance with the ethical standards of the Declaration of Helsinki (1964).

### Protocol

The occurrence of psychiatric symptoms was considered on the basis of the Diagnostic and Statistical Manual of Mental Disorders (21). Psychosocial variables of patients were collected by clinical interviews. The perception of disability in the domains of interaction with others and participation in the community activities was assessed with the World Health Organization Disability Assessment Schedule 2.0 (WHODAS 2.0) (22). The clinical assessment was based on a protocol that includes the Disability Score (DS) (23) as a global measure of the disability; the GMFCS-E&R (18), the Manual Ability Classification System (MACS) (24), and the Communication Function Classification System (CFCS) (25) for the measure of limitations for motor, manual, and communication functioning.

The parental style was estimated by the physician in association with the therapists on the basis of the clinical interviews collected during the in-charge time.

#### Psychiatric symptoms

The occurrence of psychiatric disorders was retrieved by a physician on the basis of the clinical records by using the Diagnostic and Statistical Manual of Mental Disorders, Fifth Edition (DSM-5). The occurrence of the following disorders was considered: anxiety disorders, disruptive behavior disorders, depressive disorders, obsessive-compulsive disorders, and psychotic spectrum disorders. Moreover, the following combinations of symptoms were considered: depression with anxiety and obsessive-compulsive disorders with depression.

#### Psychosocial variables

Sex, age, marital and living status, and medications at the time of the interview were considered psychosocial factors. The marital status was defined as uninvolved (unmarried/divorced) or involved (married/with a partner). The living status was defined as living with a family (the family of origin/with the acquired family) or alone. The assumption of the following medications was considered: for the treatment of seizures, anti-depressant, anti-spastic, anti-anxiety, and anti-psychotic drugs. The duration of treatment for inpatients in our institute was also considered.

#### Perception of disability

The WHODAS 2.0 Italian version was used to address the perceived disability in the domains of *getting along with others* and *participation in society*. The WHODAS 2.0 Italian version is a 36-item ICF-based generic disability assessment, which addresses disability in terms of the difficulties experienced by an individual in the last month due to a health condition. Answers are rated on a five-point scale, from no problems to complete problems/cannot do the activity. Both total and subscale scores are available, ranging from 0 to 100, with higher scores reflecting greater disability.

#### Clinical factors

##### Intellectual disability

The occurrence of an ID was determined when the Intellectual Quotient (IQ) was equal to or <70.

##### Disability score

The DS reflects the clinicians' perception of overall disability and it has been calculated according to Blair et al. (23). It derives from the sum of the scores assigned to the following disabilities: the extent and severity of body impairment, the level of cognitive impairment, and the occurrence of complications. It ranges from 1 to 12 (1–5 mild; 6–8 moderate; and severe ≥ 9). The score based on the extent of body impairment can be 1 (unilateral), 2 (bilateral mainly lower limbs), or 3 (bilateral other). The score based on the severity of body impairment can range from 0 (minimal) to 3 (severe), which is similar to the one based on ID as defined by the (IQ): 0, no ID; 1 (mild) if the IQ range is 50–69; 2 (moderate), if the IQ range is 35–49; and 3 (severe) if the IQ is below 35. The occurrence of epilepsy, blindness, and deafness may increase the global score from 0 to 3.

##### GMFCS-E&R

The GMFCS is a 5-level classification system that describes the gross motor function of children and youth with CP. Level I indicates that the patient performs gross motor skills with minimum limitations (on speed, balance, and coordination), and it represents the highest level of functioning. Level V indicates that children and youth have severe limitations in head and trunk control and require extensive-assisted technology and physical assistance.

##### The manual ability classification system

The MACS is a 5-level classification system that describes the ability of the use of hands to handle objects in everyday activities in children and youth with CP. Level I indicates that a patient is able to handle objects easily and successfully. Level V is used when the patient cannot handle objects and, due to patient's severely limited ability, needs total assistance.

##### The communication function classification system

The CFCS is a 5-level classification system that describes everyday communication performance in children and youth with any disability. Level I indicates that the patient is able to communicate easily both as sender and receiver with most people in most environments. Level V indicates that a patient is seldom effective to communicate even with familiar conversational partners.

For each scale, patients were divided into three groups: level I, level II–III, and level IV–V.

#### Parental style

The concept of parental style, based on Baumrind's studies (26), can be defined as the preferred mode of interaction that occurs between parents and children. The authors demonstrated that it has a relevant role in determining the basis for the future emotional development of the infant (27). There are four main patterns of parental style (28), characterized by different levels of care and control: authoritative, permissive, authoritarian, and neglectful. Authoritative and permissive, providing a high level of care, are the most functional types for the future state of mental health. Authoritarian and neglectful are less frequent and entail the lowest level of care with a variable level of control. These two types of parental style are related to higher rates of psychiatric symptoms, such as anxiety, depression, and dysfunctional behavior (29–31).

The parental style was estimated by the physician in collaboration with the therapists on the basis of the clinical interviews conducted during the treatment of each participant while inpatients in our institute. It was classified as dysfunctional (authoritarian or neglectful) or functional (authoritative or permissive).

### Data analysis

Descriptive statistics, i.e., frequency and percentages for categorical variables and the median and interquartile range for continuous variables, were employed to describe variable distributions. Comparisons between the psych group and the no psych group were performed using the chi-square for discrete variables and the Mann-Whitney U test for continuous variables.

We initially ran 14 separate logistic regression analyses in which the occurrence of psychiatric disorders was entered as the target variable and sex, age, marital status, living status, drugs, duration of treatment, WHODAS 2.0 (both getting along with others and participation in society scores), ID, DS, GMFCS, MACS, CFCS, and parental style as separate predictors. Since there is no cutoff, both the WHODAS 2.0 scores were collapsed into two groups considering the median value as cutoff.

We then ran a logistic regression analysis that included only those variables that were significant predictors in the previous analyses. The significance level was obtained with a *p* < 0.05 and a CI of 95%.

## Results

### Psychiatric symptoms

Psychiatric disorders were present in about one-third of the group (*n* = 59) and were distributed as follows: anxiety disorders 33.8% (*n* = 20), psychotic 25.4% (*n* = 15), disruptive disorders 16.0% (*n* = 10), obsessive-compulsive 11.9% (*n* = 7), depression 5.1% (*n* = 3), depression + anxiety 5.4% (*n* = 3), and obsessive-compulsive + psychotic 1.7% (*n* = 1).

### Psychosocial variables

Data concerning psychosocial characteristics and the comparisons between the *Psych group* and the *No psych group* of patients are presented in Table 1. The two groups differed in age (*p* = 0.005), living status (*p* = 0.04), and use of drugs (*p* < 0.001).

**Table 1:** Psychosocial characteristics (frequency and percentage) by the presence of psychiatric symptoms and results of comparisons between groups (the chi-square was used for discrete variables and the Mann-Whitney U test for continuous variables).

**Psychosocial characteristics**	**Psych group** **(*n* = 59)**	**No psych group** **(*n* = 140)**	**Comparison psych and no psych group**
**Sex**			
Male	40 (67.8%)	80 (57.1%)	**χ** ^**2**^(1) = 0.72, *p* = 0.16
Female	19 (32.2%)	60 (42.9%)	
**Age**	35 (29–42)	30 (23–40)	**U** **=** **3,079.00, Z** **=** **−2.83**, ***p*** **=** **0.005**
**Marital status**			**χ^2^(1) = 0.72, *p* = 0.39**
Uninvolved	56 (91.4%)	128 (94.9%)	
Involved	3 (8.6%)	12 (5.1%)	
**Living status**			**χ^2^(1) = 4.12, *p* = 0.04**
With the family of origin	44 (74.6%)	121 (86.4%)	
Other	15 (25.4%)	19 (13.6%)	
**Drugs**			**χ^2^(1) = 18.5, *p* < 0.001**
No	3 (5.1%)	48 (34.3%)	
Yes	56 (94.9%)	92 (65.7%)	
**Duration of treatment**	16.7 (3.4)	16.2 (4.9)	U = 3,709, Z = −1.13, *p* = 0.26

Bold values are significant, *p* < 0.05.

### Perception of disability

Both groups perceived more disability in the domain of *getting along with others* than in the domain of *participation in society*. Scores concerning the two groups are presented in Table 2. The *Psych group* complained more difficulty when compared to the *No psych group* both in the *getting along with others* (*p* = 0.014) and in the *participation in society* (*p* = 0.019) domains.

**Table 2:** Data concerning the perception of disability in two domains of the WHODAS 2.0 (median and interquartile range) and results of comparisons between groups (the Mann-Whitney U test).

**Perception of disability**	**Psych group** **(*n* = 59)**	**No psych group** **(*n* = 140)**	**Comparison psych and no psych group**
Getting along with others	33.3 (16.6–41.6)	20.83 (16.6–33.3)	**U** = **3,264.00, Z** = −**2.46**, ***p*** = **0.014**
Participation in society	25.5 (8.3–52.1)	12.5 (4.16–33.3)	**U** = **3,229.00, Z** = −**2.35**, ***p*** = **0.019**

Bold values are significant, *p* < 0.05.

### Clinical factors

Data concerning the ID, the DS severity, the type of CP, the GMFCS-E&R, the MACS, and the CMFCS and comparisons between the *Psych group* and the *No psych group* of patients are presented in Table 3. A difference was emerged in the DS severity (*p* < 0.001) and in the GMFCS-E&R (*p* < 0.05).

**Table 3:** Clinical characteristics of the whole group of patients. The chi-square was used for comparisons between groups.

**Clinical factors**	**Psych group** **(*n* = 59)**	**No psych group** **(*n* = 140)**	**Comparison psych and no psych group**
**Intellectual disability**			
Yes (IQ ≤ 70)	38 (64.4%)	79 (56.4%)	χ ^2^(2) = 1.09, *p* = 0.30
No	21 (35.6%)	61 (43.6%)	
**DS severity**			
Mild	17 (28.8%)	65 (46.4%)	**χ ^2^(2) = 6.37, *p* < 0.001**
Moderate	23 (39.0%)	34 (24.3%)	
Severe	19 (32.2%)	41 (29.3%)	
**CP subtype**			
Quadriplegia	34 (57.6%)	79 (56.4%)	χ ^2^(2) = 0.751, *p* = 0.69
Diplegia	18 (30.5%)	38 (27.1%)	
Hemiplegia	7 (11.9%)	23 (16.4%)	
**GMFCS-E&R**			
I	1 (1.7%)	16 (11.4%)	**χ ^2^(2) = 5.77, *p* < 0.05**
II–III	27 (45.8%)	66 (47.1%)	
IV–V	31 (52.5%)	58 (41.4%)	
**MACS**			
I	9 (15.3%)	44 (31.4%)	**χ** ^**2**^(2) = 5.75, *p* = 0.5
II–III	32 (54.2%)	58 (41.4%)	
IV–V	18 (30.5%)	38 (27.1%)	
**CMFCS**			
I	27 (45.8%)	64 (45.7%)	**χ** ^**2**^(2) = 0.08, *p* = 0.9
II–III	18 (30.5%)	45 (32.1%)	
IV–V	14 (23.7%)	31 (22.1%)	

Data are reported as frequency and related percentages. DS, disability score; GMFCS-E&R, Gross Motor Functioning Classification Scale Expanded & Revised; MACS, Manual Ability Classification Scale; CFCS, Communication Functioning Classification Scale. Bold values are significant, *p* < 0.05.

### Parental style

The parental style was differently distributed between the two groups, χ^2^(1) = 3.93, *p* = 0.05. A dysfunctional style was observed for 15.3% of the patients with psychiatric disorders whereas this rate was reduced to 6.4% in the group with no psychiatric disorders.

### Logistic regression

Results of logistic regression analyses are presented in Table 4. The following variables were emerged as significant predictors: age, living status, drugs, WHODAS 2.0 scores, DS severity, GMFCS, and MACS.

**Table 4:** Results of logistic regression.

**Variables**	**Odds ratio**	**Estimate (95%CI)**
**Sex**	0.66	0.33–1.20
**Age**	1.05	1.01–1.09^*^
**Marital status**		
Single	Ref	
Married	0.57	0.15-2.1
**Living status**		
With the family of origin	Ref	
Other	2.17	1.02–4.64^*^
**Drugs**	9.74	2.9–32.8^*^
**Duration of treatment**	1.04	0.96–1.13
**Whodas 2.0 getting along with others**	1.98	1.07–3.67^*^
**Whodas 2.0 participation in society**	1.02	1.00–1.03^*^
**Intellectual disability**	1.40	0.74–2.62
**DS severity**		
Mild	Ref	
Moderate	2.59	1.22–5.49^*^
Severe	1.77	0.82–3.8
**GMFCS**		
I	Ref	
II–III	6.55	0.82–51.8
IV–V	8.55	1.08–67.1^*^
**MACS**		
I	Ref	
II–III	2.7	1.17–6.23^*^
IV–V	2.3	0.93–5.75
**CFCS**		
I	Ref	
II–III	0.95	0.47–1.92
IV–V	1.07	0.49–2.32
**Parental style**		
Functional	Ref	
Dysfunctional	2.62	0.98–6.98

* p < 0.005.

A final stepwise regression identified five significant predictors of the occurrence of psychiatric disorders (see Table 5): living status, drugs, parental style, and WHODAS 2.0 getting along with others score (all *p*'s < 0.005). The AIC (Akaike information criterion) of the overall model was 215. Receiver operating characteristic (ROC) curve shows that the predictive accuracy within the logistic regression model was satisfactory [area under the curve (AUC) = 0.75].

**Table 5:** Summary of the logistic regression.

**Variables**	**Estimate (95%CI)**	**Odds ratio**	**95% CI**
Living status	1.14	3.14	1.28–7.75
Drugs	2.32	10.22	2.92–35.8
Parental style	1.38	4.00	1.23–12.93
Whodas 2.0 getting along with others	0.83	2.13	1.10–4.81

All p's < 0.005.

## Discussion

This paper explored the occurrence of psychiatric symptoms in adults with CP aged 20–50. About one-third of the samples had at least one psychiatric symptom. This result is similar to McMorris et al. (11) who found a prevalence of 33.7% of any psychiatric disorder in patients with CP, as well as in Peterson et al. (8) who identified an incidence of 38.8% of any psychological morbidity in these patients. In the present study, the more frequently observed disorders were anxiety and psychotic disorders, followed by disruptive, obsessive-compulsive disorder, and depression. Psychotic and disruptive disorders occurred four-fold in patients with a concurrent ID. This ratio was less skewed when considering anxiety as it occurred with a 3–2 ratio in patients with ID. This is similar to McMorris et al. (11), Jonsson et al. (16), and Whitney et al. (9) who showed a prevalence of psychotic disorders in patients with CP and ID and a prevalence of anxiety disorders in patients with only CP.

Gender when accounted for psychosocial variables did not affect the rate of psychiatric disorders. This appears in disagreement with Axmon et al. (32), Taggart et al. (33), and Tsakanikos et al. (34) who instead found gender differences with respect to several types of psychiatric diagnoses. This difference might be explained as we did not consider each psychiatric disorder separately, as these authors did. Aging increased the occurrence of psychiatric disorders. Since in this sample, anxiety was the most represented disorder, the observed impact of age seems partially in accordance with Kessler et al. (35). Authors found that the age of onset of anxiety occurred later (end of the 20s and until middle 70s) than other presentations, similar to Whitney et al. (17) who found an increase in the prevalence of anxiety from ages 18–30 years to ages 31–40 years. Most of the participants were uninvolved in a relationship and no differences were observed between groups. On the other hand, living status was a relevant predictor of psychiatric symptoms. Patients who lived outside the family of origin (alone, in an institution, or with an acquired family) had triple odds to have a psychiatric disorder, especially those patients who lived alone more frequently than other patients (75 vs. 25%, respectively).

The assumption of any drug had increased about eight times the odds of having a psychiatric disorder. In this sample, the anti-epilepsy drugs and the anti-spastics were the most frequently assumed medications (from 20 to 38% of assumption, respectively). About one patient out of ten used anti-anxiety, anti-depressants, or anti-psychotics. The observation that about three-quarters of participants assumed medications is in accordance with Pons et al. (36) who found that patients with CP took a large number of different types of drugs. Partially in agreement with our results, Pons et al. found that the most frequent conditions addressed by medications were epilepsy, psychiatric disorders, and spasticity. In our sample, about 30% took drugs for a psychiatric condition only, but this rate could be even larger because we did not include those used for epilepsy and that can be used also to treat psychiatric disorders. In particular, the use of drugs for the treatment of epilepsy is more frequent in patients with quadriplegia (37).

A greater perception of disability both in the participation in society and getting along with others increased the odds of a psychiatric disorder. Whitney et al. (3) found that social factors, such as difficulties in friendships and lower participation in activities, were associated with a higher prevalence of mental health disorders since childhood in patients with CP.

Patients with a moderate global disability had an increased odd of showing psychiatric disorders than patients with a severe disability. This could be in accordance with Parkes et al. (38) who found that differences in functional ability were more stressful and therefore may have a greater psychological impact on children with CP when they were more similar to their able-bodied peers than when these differences were more evident. In accordance with Rackauskaite and Bjorgaas, the type of CP was not associated with the occurrence of psychiatric symptoms (4, 7).

From a clinical point of view, having a severe motor impairment, that is scoring GMFCS levels IV and V or having a moderate manual impairment, that is MACS levels II or III, both increased the risks of psychiatric disorders when compared to patients in level I. This did not occur for the communication abilities. Data related to the prevalence of mental health symptoms depending on GMFCS, CFCS, or MACS levels are contradictory. Jarl et al. (14) observed an increase in all problems (that include anxiety and depression) in a sample of adults with CP when lower functioning in terms of GMFCS, MACS, and CFCS was reported. Similarly, Van der Sloot et al. (12) found that adults with CP who scored GMFCS level III or IV had more depressive symptoms than adults who scored level I or II. In addition, Van Gorp et al. (15) showed that patients with GMFCS I had fewer depressive symptoms when compared to the age-matched reference population. By contrast, Jonsson et al. (16) when compared different levels of GMFCS and CFCS found no significant differences in psychiatric symptoms, depression, and anxiety. Our results, therefore, are in agreement with those that showed an increased risk of psychiatric disorders when a severe motor impairment is present. The limitations on mobility and physical activities lead to difficulties in engaging with social relationships, exclusion, and social isolation, as suggested by McMorris et al. (11), which could reasonably have a role in increasing the risk of psychiatric disorders.

Growing in a family with a dysfunctional parenting style increased the odds of having a psychiatric disorder in adulthood by more than four time. Several studies demonstrated an association in healthy people between a dysfunctional parental style and the occurrence of many forms of mental disorders (29, 30, 39), as well as chronic pain (40). In this sample, patients who lacked adequate care as often occurs in the neglectful and authoritarian parental style have more chances to have psychiatric symptoms. This result is in accordance with Enns et al. (29) who found that lack of care was the parenting variable most consistently related to a wide variety of forms of adult psychopathology.

The significant predictors in the final logistic regression were living status, drugs, parental style, and the score in the “getting along with others” scale of WHODAS 2.0. This model has a high specificity (96%) but a lower sensitivity (23%), which means that by considering these predictors, the model correctly predicts those patients with CP without psychiatric disorders in 96% of cases but predicts only 23% of patients who have a psychiatric disorder.

The primary limitation of this study is that it included only patients who were under a single Institution in a specific territory. In addition, the occurrence of psychiatric symptoms and the type of parental style were not identified by using a structured instrument, therefore, could be underestimated.

A wide sample of adult patients with CP was involved in this study. Results suggested that some clinical and psychosocial variables should be kept in mind during rehabilitation as potentially influencing the mental health evolution in these patients and in the long run their final functioning outcomes.

## Data availability statement

The raw data supporting the conclusions of this article will be made available by the authors, without undue reservation.

## Ethics statement

The studies involving human participants were reviewed and approved by Ethics Committee (Prot. N. 61/17—CE). The patients/participants provided their written informed consent to participate in this study.

## Author contributions

SPiz and AP collected, coordinated, and supervised data collection. SPiz and MU drafted the initial manuscript and reviewed and revised the manuscript. MM, SPic, and MV drafted the initial manuscript. AM conceptualized and designed the study and reviewed and revised the manuscript. All authors approved the final manuscript as submitted and agree to be accountable for all aspects of the work.

## Funding

This research was supported by the Italian National Institutes of Health (Grant RC 1018001).

## Conflict of interest

The authors declare that the research was conducted in the absence of any commercial or financial relationships that could be construed as a potential conflict of interest.

## Publisher's note

All claims expressed in this article are solely those of the authors and do not necessarily represent those of their affiliated organizations, or those of the publisher, the editors and the reviewers. Any product that may be evaluated in this article, or claim that may be made by its manufacturer, is not guaranteed or endorsed by the publisher.
